# Classification of Botrytized Wines Based on Producing Technology Using Flow-Modulated Comprehensive Two-Dimensional Gas Chromatography

**DOI:** 10.3390/foods10040876

**Published:** 2021-04-16

**Authors:** Olga Vyviurska, Nemanja Koljančić, Ha Anh Thai, Roman Gorovenko, Ivan Špánik

**Affiliations:** Faculty of Chemical and Food Technology, Institute of Analytical Chemistry, Slovak University of Technology in Bratislava, 81237 Bratislava, Slovakia; olga.vyviurska@stuba.sk (O.V.); nemanja.koljancic@stuba.sk (N.K.); ha.thai@stuba.sk (H.A.T.); romagorovenko@gmail.com (R.G.)

**Keywords:** enantioselective analysis, flow-modulated comprehensive two-dimensional gas chromatography, botrytized wines, Tokaj wine region

## Abstract

The enantiomeric ratio of chiral compounds is known as a useful tool to estimate wine quality as well as observe an influence of wine-producing technology. The incorporation of flow-modulated comprehensive two-dimensional gas chromatography in this type of analysis provides a possibility to improve the quality of results due to the enhancement of separation capacity and resolution. In this study, flow-modulated comprehensive two-dimensional gas chromatography was incorporated in enantioselective analysis to determine the influence of winemaking technology on specific features of botrytized wines. The samples included Tokaj essences (high-sugar wines), Tokaj botrytized wines and varietal wines (Furmint, Muscat Lunel, Lipovina) and wines maturated on grape peels. The obtained data was processed with hierarchic cluster analysis to reveal variations in composition and assess classification ability for botrytized wines. A significant difference between the samples was observed for the enantiomeric distribution of ethyl lactate and presence of monoterpene alcohols. The varietal wines were successfully separated from the other types, which showed more similar results and could be divided with additional parameters. We observed a correlation between the botrytized wines and the varietal wines fermented with grape skins. As to the essences produced from juice of botrytized grapes, the results were quite similar to those of the botrytized wines, even though monoterpenes were not detected in the extracts.

## 1. Introduction

The pleasant honey-like taste and unique fruit flavor of botrytized (noble rot) wines are the result of a specific winemaking technology, which includes overripe grapes infected by *Botrytis cinerea*. The fungus induces increased content of sugar and fatty acid aroma precursors, and the formation of new compounds in grapes [1]. According to Schmitt-Kopplin et al. [2], Botrytis infection initiates fermentation retardation of the yeast metabolomics activity during alcoholic fermentation of wine. The distinctive climate condition of Tokaj wine region (soil slopes of volcanic origin and surrounding wetlands) supports growth of *Botrytis cinerea* on grapes [3]. Furmint, Muscat Lunel and Lipovina are the main grape varieties in the region for production of wine specialities and dry white wines. For example, Tokaj essence is made as juice of botrytized berries obtained by gravitation during harvest season [4]. High sugar content (65 to 752 g/L) supports long term-fermentation resulting in 5–7% alcohol [5]. At the same time, aging of the essence increases the concentration of polyphenols and its antioxidant properties [6]. In the case of the botrytized wines, infected berries are picked up and macerated in grape must for one or two days. A ratio of noble-rotten grape to grape (tub, “putňa” in Slovak, “puttonyos” in Hungarian) determines the sweet, smooth taste, and pleasant aroma of the wine. One “putňa” includes one barrel of noble-rotten grape berries (20–25 kg) per 136–140 L of one-year-old young wine. Tokaj botrytized wines are commonly aged from 3 to 5 years in oak barrels. A more detailed description of winemaking technologies and classification of botrytized wines from Tokaj wine region is reported by [7]. As for the volatile organic compounds, significant difference in volatile ethyl esters, fatty acids and sherry lactones, was revealed for botrytized Amarone wine in comparison to the same wine produced from healthy grapes [8]. Furthermore, an increase in 3-sulfanylhexan-1-ol was reported for in the botrytized wines produced from Sauvignon blanc and Semillon grapes [9]. A dominance of S-3-sulfanylhexan-1-ol was typical for these botrytized wines, whereas a racemic ratio was observed for dry white Sauvignon blanc and Semillon wines [10].

Chirality is one of the most important properties of organic compounds included in the composition of food products [11]. The importance of determining enantiomers and their ratio lies on the fact that enantiomers volatile molecules have different aroma characteristics and odor detection threshold [12]. Changes in enantiomeric ratio could yield information about product quality, technical processing, different biological activity, and contamination [13]. In some cases, discrimination of samples can be problematic due to an additional racemization of chiral compounds through fermentation or distillation processes. Thus, a complex approach with a number of enantiomeric data and chemometric modelling has been demonstrated in the recent studies. For example, the enantiomeric concentrations of terpenes and (1R, 2R)-methyl jasmonate were exploited in statistics discrimination of 22 tea cultivars according to their geographical origin [14]. Influence of different storage conditions and manufacturing process on green tea was shown with a total distribution of catechins and methylxanthines [15]. The PCA analysis was included in the metabolomics profiling of chiral amino acids for classification of cheese depending ripening period (6, 18, 26 months) [16]. Castells et al. [17] proposed to discriminate honey origin with the enantiomeric ratio of dintitrophenyl amino acids. The authors [18] developed a chemometric method based on the composition of triacylglycerols and volatiles for varietal classification of extra virgin olive oils. Comprehensive two-dimensional gas chromatography (GC×GC) shows clear advantages for the analysis of complex samples, such as improved separation capacity, increased number of identified compounds, structured chromatograms and significant signal enhancement [19]. Enantioselective GC×GC analysis was successfully used for the evaluation of essential oils [20,21] and herbal products [22]. Flow-modulated comprehensive two-dimensional gas chromatography proposed more available equipment as well gives a possibility to adjust amount of sample directed to the second column. In our study, we tried to exploit enantioselective GC×GC analysis for the evaluation of botrytized wines in comparison to the corresponding varietal grape wines, selected essences, and the varietal wines fermented with grape skins. 

## 2. Materials and Methods

### 2.1. Chemicals 

n-Hexane and standards of enantiomers were supplied from Sigma Aldrich (St. Louis, MO, USA), and sodium chloride was obtained from Chemapol (Prague, Czech Republic). A mixture of n-alkanes (C7-C30) used for the calculation of retention indices was purchased from Supelco (Belleforte, PA, USA).

### 2.2. Samples

The samples included Tokaj essences (ES), wine maturated on grape peel (GP30 for 30 and GP90 for 90 days), botrytized wines (2, 3, 4, 5 and 6 putňove, 2P1-6P8) and varietal wines (Furmint, Muscat Lunel and Lipovina variety, F1-L5). More detailed information about the used samples is presented in Table 1.

### 2.3. Sample Preparation

The studied samples were prepared by liquid-liquid extraction procedure using n-hexane. First, 2.0 g of sodium chloride was added into a 20 mL aliquot of wine. Then, the mixture was transferred to a separatory funnel in order to facilitate extraction. Next, 5 mL of n-hexane was added to the funnel, the mixture was shaken by hand for 5 min and extraction was repeated two more times under the same conditions. The combined extracts were centrifuged at 3600 rpm for 10 min. The resulting organic extract was evaporated to 1 mL under nitrogen flow in a 55 °C water bath. The development of the method is described in detail in [23].

### 2.4. Instrumentation

Agilent 7890A gas chromatograph (Wilmington, DE, USA) coupled with a reverse fill/flush (RFF) flow modulator (Agilent G3486A CFT Modulator, Folsom, CA, USA), flame-ionization detector (FID) and quadrupole mass spectrometer (qMS) were used to determine an enantiomer ratio in the wine samples. The GC column setup contains 30 m × 0.25 mm × 0.25 μm Rt-ßDEXse (Restek, Bellefonte, PA, USA) in the first dimension and 5 m × 0.25 mm × 0.15 μm INNOWax (Agilent Technologies, Folsom, CA, USA) in the second dimension. A supplementary restrictor (5 m × 250 µm ID) facilitates a switch of the carrier gas direction in the modulator between loading and injection mode. The modulation period was set to 6 s and included a 0.11 s sampling time. The second column effluent is directed to a splitter connected to qMS and FID detectors. Such approach is connected to compatibility of the detectors with elevated second flow of carrier gas and high acquisition frequency required for GC×GC analysis. A 0.5 m × 100 µm ID restrictor and a 1.2 m × 250 µm ID restrictor were installed to qMS and FID, respectively.

An initial temperature of the oven program was 40 °C and kept for 10 min. Further, temperature was increased with a rate 2 °C/min to 220 °C and maintained for 25 min. A total analysis time was 125 min. 1 µL of the sample extract was injected in splitless mode into 250 °C heated inlet. Helium (99.999% purity) in constant flow mode was used as the carrier gas. 0.7 mL/min flow rate was set in the first dimension and 23 mL/min for the second dimension. Flow rates to FID and MS detectors were determined with parameters of the restrictors and set as 23.3 mL/min and 2.1 mL/min, respectively. The flame-ionization detector was operated at 250 °C with a hydrogen flow rate of 30 mL/min, an air flow rate of 450 mL/min, and a makeup flow rate of 25 mL/min. A data acquisition rate of 100 Hz was used for FID detector. A transfer line to MS detector was kept at 250 °C for whole run time. Ion source temperature and quadrupole temperature were maintained at 180 °C and 300 °C, respectively. The MS signal acquisition rate was 21.43 spectra/s (40–400 m/z range). The primary processing of the obtained chromatograms was performed using GC Image software version v. 2.1. (Zoex Corporation, Houston, TX, USA), and MSD ChemStation software (version F.01.01.2317, Agilent Technologies, Santa Clara, CA, USA) with NIST14, FFNSC2, MPW2007 and W9N11 databases. GC×GC-MS identification of compounds was also supported with injection of standard compounds.

## 3. Results

**Figure 1:**
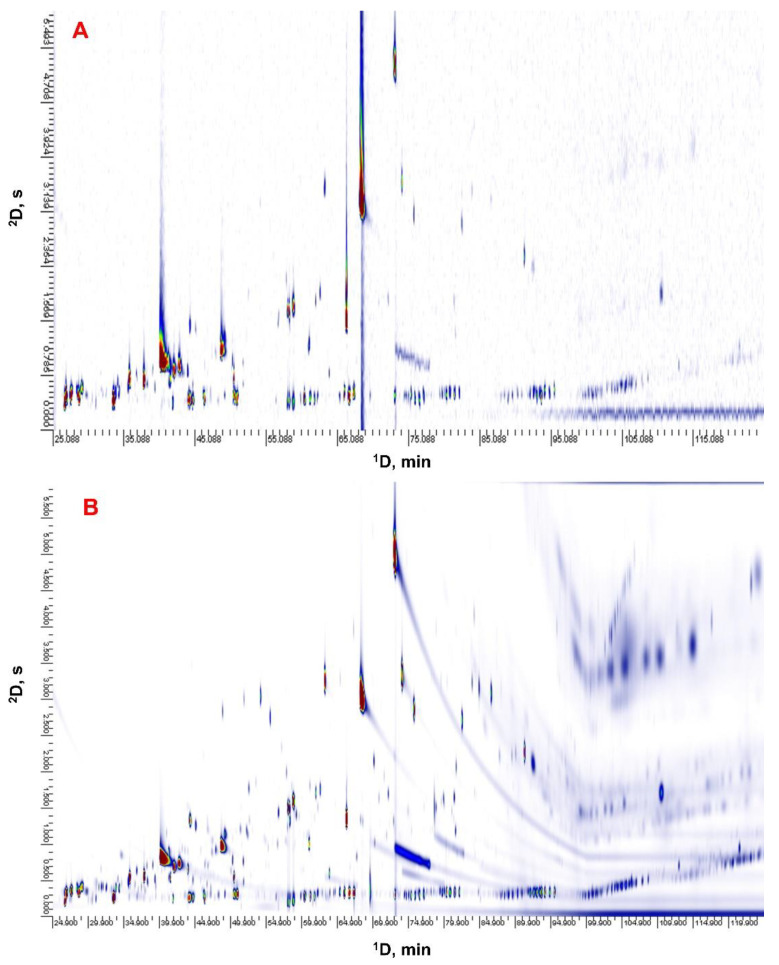
GC×GC-MS (**A**) and GC×GC-FID (**B**) chromatogram of Furmint varietal wine (2015).

A chiral column with the stationary phase based on 2,3-di-O-ethyl-6-O-*tert*-butyl dimethylsilyl-β-cyclodextrin, was used in the first dimension for GC×GC analysis. A polar INNOWax column was selected for the second dimension to separate analytes according to polarity. GC×GC-MS data was used to determine chiral volatile compounds presented in the samples. Due to higher acquisition rate of flame-ionization detector and narrower peaks, GC×GC-FID data was preferred for calculation of enantiomeric ratio. GC×GC-MS and GC×GC-FID chromatograms of Furmint varietal wine (2015) are represented in Figure 1. More detailed information about volatile organic compounds composition of Tokaj varietal wines and Tokaj selection wines can be found in the previous studies [23,24]. The target chiral compounds include ethyl lactate, linalool, α-terpineol, γ-nonalactone and whiskey lactone. Retention times and retention indices of stereoisomers are shown in Table 2. Enantiomeric ratio of the chiral compounds was estimated according to:

$$E_{R}=\frac{A_{R}}{A_{R}+A_{S}}\times 100$$

where A_R_ is obtained peak area of R enantiomer and A_S_ is obtained peak area of the following S configuration [25]. RSD values of enantiomeric ratios based on GC×GC-FID were less than 10%.

### 3.1. Ethyl Lactate

Ethyl lactate is an important aroma compound which contributes to the “broader”, “fuller” taste of wine and could be used for the determination of the microbiological infection of wine [26]. Enantiomers of ethyl lactate are obtained through different fermentation processes that are typical for winemaking. R-(+)-ethyl lactate is a product of sugar fermentation by yeast, whereas presence of S-(-)-ethyl lactate is caused by activity of lactic acid bacteria during malolactic fermentation [27]. Lactic bacteria (Lactobacillus, Pediococcus, and Oenococcus) support conversion of L-malic acid to L-lactic acid and additional biosynthesis of aroma compounds [28]. Table 3 shows that both of R- and S- enantiomers were detected in 46 from 49 samples. The exception was observed for varietal wine produced from Muscat Lunel (2016) and Lipovina (2015). Overall, R-(+)-ethyl lactate was dominant for Tokaj varietal wines in comparison to the other types. The highest value of R-(+)-ethyl lactate (91%) was detected in Furmint (2015). The excess of S-(-)-ethyl lactate over R-(+)-ethyl lactate varied from 2 times (4P-1993, 4P-2009) to 6 times (2P-1990, 3P-1999) in the botrytized wines. A few samples like 3P-2009, and 6P-2006 showed a reverse tendency with the R:S ratio as 70:30, 81:19, respectively. It is worthy to remark that higher excess of S-enantiomer (nearly 8 times) was recorded in the wine fermented with grape skins for 30 days. A content of R-enantiomer increased almost double in after 90 days of fermentation with grape skins. Mills et al. [29] showed a presence of atypical lactic acid bacteria community in botrytized wines (Leuconostoc and Lactococcus), which could be connected to Botrytis colonization on the grape berry. Increased content of S-enantiomer was shown for the other types of wine [27,30] after malolactic fermentation. For example, Freitas et al. [31] claimed a decrease of R-(+)-ethyl lactate by 50–68%, and an increase of 85–75% for S-(-)-ethyl lactate as a result of activity of lactic acid bacteria. 

### 3.2. Terpenes

**Figure 2:**
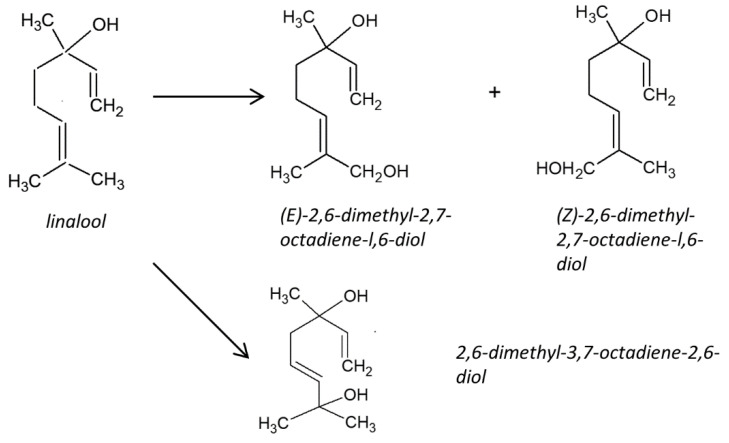
Transformation of linalool (modified [34]).

A majority of terpenes are bonded to sugar molecules and occurs in grapes in nonvolatile form. Their concentration increases during grape ripening and wine ageing [32] and become important components of wine flavor and aroma [33]. A racemic mixture of terpenes is commonly observed in raw fruits or as a product of fermentation process. In our case, linalool was mostly presented in the varietal Tokaj wines (ten out of twelve samples). For the botrytized wines, linalool was detected only in two samples, whereas other types of samples did not contain linalool at all. In particularly, only 3 and 4 “puttony” wines (both from 2009) were reported to contain linalool enantiomers. It is worthy to note that a racemic mixture was obtained in almost half of total samples, e.g., Furmint–2015 (48:52), Muscat Lunel–2015 (49:51). A slight dominance of S-stereoisomer (59–63%) was observed for some Muscat Lunel and Lipovina samples. In botrytized wines, linalool generally metabolizes to (E)-2,6-dimethyl-2,7-octadiene-l,6-diol (>95%) (Figure 2) [34]. The other biotransformation by-products include (Z)-2,6-dimethyl-2,7-octadiene-l,6-diol, 3,9-epoxy-p-meth-1-ene, furanoid (Z)- and (E)-linalool oxides, pyranoid (Z)- and (E)-linalool oxides, and 2-vinyl-2-methyl-tetrahydrofuran-5-one. Overall, linalool level tends to reduce with prolonged wine aging due to conversion to α-terpineol and furan linalool oxides [35]. For example, concentration of linalool decreased by 3.3 and 71.6 times for Alvarinho and Loureiro wines after 20 months of maturation [36].

α-Terpineol is another monoterpene alcohol which concentration correlates with ageing period of wine. Ferreira et al. [35] showed a significant increase in α-terpineol content after one week of accelerated aging, and the further decrease at the end of aging period (nine weeks). A positive effect of *Botrytis cinerea* on α-terpineol concentration in wine was found in comparison with sweet Chardonnay wines [37]. In contrast to linalool, α-terpineol was more typical for botrytized wine samples. However, the GC×GC-MS analysis did not confirm the presence of α-terpineol stereoisomers in Tokaj essences and fermented with grape skins. Interestingly, α-terpineol mostly occurs in the samples with higher “putňa” number, e.g., four samples for 6P wines vs. one sample for 3P wines. Almost racemic ratio was found in those samples and slightly higher enantiomeric ratio (64%) was obtained for S-enantiomer in 6P wine (2002). As to the varietal wines, the majority of samples contained α-terpineol as racemate. Two samples of Muscat wine showed small dominance (>60%) of R-(+)-α-terpineol. Unfortunately, the results of α-terpineol enantiomers cannot be used for the differentiation of wines according to grape variety or “putňa” number. 

### 3.3. Lactones

γ-Lactones are commonly identified in wine, where they play an important role as aroma active compounds. Organoleptic properties of lactones are mainly determined by carbon chain attached to a carbon ring [38]. In winemaking, formation of lactone could occur in grapes, through fermentation or during aging processes by their extraction from oak wood [39]. Azpilicueta et al. [39] reported that accumulation of γ-nonalactone from American oak barrels did not dependent on wine type (Merlot or Cabernet Sauvignon), and constant concentration could be achieved after two months of aging. Aroma characteristics of γ-nonalactone enantiomers are slightly different, e.g., soft coconut and sweet taste is typical for R-stereoisomer, whilst weak coconut is observed for S-stereoisomer [40]. As can be seen from Table 3, both R and S configurations could be found in all wine categories. However, γ-nonalactone was observed in less samples than ethyl lactate. In case of 3P wines, γ-nonalactone was detected in 6 out of 7 samples. Whereas for the other botrytized wines, this compound was presented in a half of the samples. The similar results were observed for the varietal wines (7/12). Overall, R-γ-nonalactone is dominant (58–80%) for all the samples. The similar findings were also reported for Australian botrytized white wines [40] and Bordeaux dessert wines [41]. As can be seen from the results for the wines fermented with grape skins, fermentation period did not significantly affect stereoisomeric distribution. 

Originally, whiskey (oak) lactone was identified as *cis*- and *trans*-5-n-butyl-4-methyl-4,5-dihydro-2(3H)-furanone in burbon whiskey [42]. Whiskey lactone molecule has four stereoisomers, but only *trans*-(3S,4R)-and *cis*-(3S,4S)-whiskey lactones are naturally occurring [43]. Although *cis*-stereoisomer has a lower odor threshold, both *cis*- and *trans*-whiskey lactones contribute to fresh wood and coconut aroma of wines [44]. A number of factors influences a degree of whiskey lactone extraction from oak barrels, e.g., composition of wood, toasting processes, and wine ageing period in barrels [45]. At higher concentration, whiskey lactones can become a predominant flavor compound in wines. This problem can occur in new winery where new wood barrels are used for wine ageing or if wine maker is not careful and large amount of whiskey lactones is extracted from wooden barrels [46]. The two main oak species (French oak and American oak) are traditionally used for wine aging. It was shown that American oak releases particularly *cis*-whiskey lactone, especially with new oak barrels [47]. A *cis*/*trans* ratio of whiskey lactone has been suggested as a parameter to distinguish wines aged in American or French oak barrels. For example, Alamo-Sanza et al. [48] found that the content of *cis*-whiskey lactone is 5-fold greater higher than the content of *trans*-enantiomer for wines aged in American oak barrels, whereas for French oak barrels this value was only doubled. Nearly 10% of *trans*-stereoisomer was detected in wine after aging in American oak [49], and almost racemic mixture was measured in the case of French oak. In our case, almost all botrytized samples contain whiskey lactone, except of one sample produced 1990. The reversed situation was observed for the varietal wines, where whiskey lactone was detected in two samples from Furmint and Lipovina (2015). Overall, *cis*-whiskey lactone dominated in the samples (52–73% range), and a *cis*/*trans* ratio varies from 1.1 to 2.7. Interestingly, this correlation between stereoisomers was higher (3.2) for the wine fermented with grape skins 30 days, and it decreased to 1.9 value after 90 days. The similar enantiomeric distribution (62% *cis*-whisky lactone) was shown for essences, which undergo long-term aging. 

### 3.4. Hierarchical Clustering Analysis of the Wine Samples

Clustering analysis is commonly used to determine data structure and look for similarities between multiple objects [50]. It has been successfully incorporated in food analysis to emphasize bioactive components and functional properties of products [51]. In order to check the significance of variations in the target compounds composition, hierarchical cluster analysis was selected as a classification tool. The ratios for R-(+)-ethyl lactate, R-(-)-linalool, R-(+)-α-terpineol, R-γ-nonalactone, *cis*-whiskey lactone were included in the calculations. The data obtained for the botrytized wines were averaged to simplify a dendrogram. Distances between the samples were estimated with Euclidean distances by the following formula:

$$d=\sqrt{{\left(x_{1}-y_{1}\right)}^{2}+{\left(x_{2}-y_{2}\right)}^{2}+\ldots +{\left(x_{n}-y_{n}\right)}^{2}},$$

where $x_{1},x_{2}\ldots x_{n}$ and $y_{1},y_{2}\ldots y_{n}$ represents co-ordinates of two points in *n*-dimensional space. A distance between two clusters was determined by the distance of the furthest neighbours in two clusters. This approach called complete linkage is recommended to decrease number of undistinguished clusters. 

**Figure 3:**
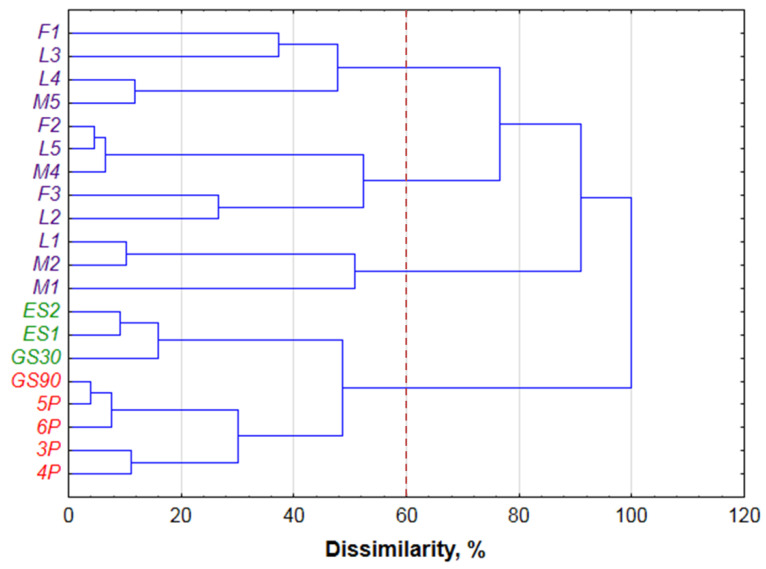
HCA dendrogram of the obtained data. Dissimilarity is calculated as a ratio of d_link_ to d_max_.

The dendrogram in Figure 3 illustrates the stages of linkages and reveals successful separation of the samples on the varietal wines and the others. Unfortunately, it was not possible to classify the varietal wines accordingly to grape variety, and three groups are clustered based on the analysed variables. From other side, with a few exceptions (L2 and L4), some dependence from wine producer could be supposed. The first group (F1, L3, L4, M5) contains the samples supplied from Tokaj & Co (Malá Tŕňa, Slovakia, whereas the varietal wines from Ostrožovič (Veľká Tŕňa, Slovakia) mainly belong to the second (F2, L5, M4, F3, L2) and the third (L1, M1, M2) groups. This assumption requires a larger number of samples or the incorporation of additional variables to be confirmed. 

As for the other samples, this group is divided on two subgroups with essences and botrytized wines. It is worthwhile noting, that influence of period of fermentation period with grape skins was confirmed with cluster analysis. The sample obtained after 30 days of fermentation (GP30) was sorted to essences, and the sample with prolonged fermentation (90 days) was more similar to the botrytized wines.

## 4. Conclusions

The results obtained through chiral analysis with flow-modulated comprehensive two-dimensional gas chromatography, show a significant difference between wine categories. Particularly, it can be seen for the varietal wines and the botrytized wines, where dissimilarity is also confirmed with hierarchic cluster analysis. In this case, the variations in data are mainly related to ethyl lactate, linalool and whiskey lactone. The dominance of S-(-)-ethyl lactate in the botrytized wines is supposed to be a result of malolactic fermentation supported by *Botrytis cinerea* colonization on the grape berry. Another finding which could correlates with the influence of the fungus on winemaking technology is the low presence of monoterpene alcohols (especially linalool) in comparison with the varietal wines. R-γ-nonalactone prevails in all the samples, whereas a content of whiskey lactone is directly connected to wine aging condition. The essence samples belong to a special high-sugar wine category and obtained from juice of from botrytized berries, show similar results to the botrytized wines. Moreover, monoterpenes were not observed at all in the extracts. Interestingly, that the enantiomeric distribution of the target compounds changes with simultaneous fermentation of the varietal wines with grape skins. According to cluster analysis the sample is classified to an essence subgroup after 30 days of fermentation. In the case of 90 days of fermentation, the results were more comparable with the botrytized wines. Increased enantiomeric ratio of R-ethyl lactate and a reduction in a *cis*/*trans* ratio of whiskey lactone are observed with the extension of the fermentation period.

## Figures and Tables

**Table 1:** The samples used for investigation.

Code	Year	Producer	Code	Year	Producer
ES1	1999	TOKAJ & CO.	5P7	2000	Zlatý Strapec
ES2	2000	J & J OSTROŽOVIČ	5P8	2003	J & J OSTROŽOVIČ
GP30	2016	J & J OSTROŽOVIČ	5P9	2003	J & J OSTROŽOVIČ
GP90	2016	J & J OSTROŽOVIČ	5P10	2004	J & J OSTROŽOVIČ
2P1	1989	TOKAJ & CO.	6P1	1977	Zlatý Strapec
2P2	1990	TOKAJ & CO.	6P2	1983	Zlatý Strapec
3P1	1988	Zlatý Strapec	6P3	1989	J & J OSTROŽOVIČ
3P2	1990	TOKAJ & CO.	6P4	1989	TOKAJ & CO.
3P3	1995	J & J OSTROŽOVIČ	6P5	1999	J & J OSTROŽOVIČ
3P4	1995	Zlatý Strapec	6P6	2002	J & J OSTROŽOVIČ
3P5	1999	J & J OSTROŽOVIČ	6P7	2003	J & J OSTROŽOVIČ
3P6	2000	Zlatý Strapec	6P8	2006	TOKAJ & CO.
3P7	2009	TOKAJ & CO.	F1	2014	J & J OSTROŽOVIČ
4P1	1993	Zlatý Strapec	F2	2015	TOKAJ & CO.
4P2	1995	TOKAJ & CO.	F3	2015	J & J OSTROŽOVIČ
4P3	2000	Zlatý Strapec	M1	2016	J & J OSTROŽOVIČ
4P4	2002	J & J OSTROŽOVIČ	M2	2016	J & J OSTROŽOVIČ
4P5	2004	J & J OSTROŽOVIČ	M4	2015	J & J OSTROŽOVIČ
4P6	2009	TOKAJ & CO.	M5	2015	TOKAJ & CO.
5P1	1959	Zlatý Strapec	L1	2015	J & J OSTROŽOVIČ
5P2	1972	Zlatý Strapec	L2	2015	TOKAJ & CO.
5P3	1989	J & J OSTROŽOVIČ	L3	2015	TOKAJ & CO.
5P4	1990	TOKAJ & CO.	L4	2015	J & J OSTROŽOVIČ
5P5	1993	J & J OSTROŽOVIČ	L5	2015	J & J OSTROŽOVIČ
5P6	1993	Zlatý Strapec	-	-	-

2P, 3P, 4P, 5P and 6P are shortcut for “puttony” wines with the corresponding number of tubs was added.

**Table 2:** Target chiral compounds.

Compounds	RT1, min	RT2, s	RI	Resolution
R-(+)-ethyl lactate	44.288	1.491	972	-
S-(-)-ethyl lactate	44.988	1.491	979	3.29
R-(-)-linalool	60.388	1.177	1204	-
S-(+)-linalool	60.688	1.177	1210	1.56
R-(+)-α-terpineol	68.288	1.491	1322	-
S-(-)-α-terpineol	68.588	1.491	1328	2.59
*cis*-whiskey lactone	78.788	1.648	1506	-
*trans*-whiskey lactone	81.488	1.805	1543	11.00
R-γ-nonalactone	84.388	1.833	1592	-
S-γ-nonalactone	84.588	1.833	1595	8.20

RT1 corresponds to retention time of compounds eluted from first dimension and RT2 corresponds to retention time of compounds eluted from second dimension, RI—retention index for the 1st column.

**Table 3:** Results of GC×GC-FID analysis.

Code	Enantiomer Ratio [%]									
	R-(+)-Ethyl Lactate	S-(-)-Ethyl Lactate	R-(-)-Linalool	S-(+)-Linalool	R-(+)-α-Terpineol	S-(-)-α-Terpineol	R-γ-Nonalactone	S-γ-Nonalactone	*trans*-Whiskey Lactone	*cis*-Whiskey Lactone
**Essences**										
ES1	24	76	-	-	-	-	61	39	38	62
ES2	30	70	-	-	-	-	74	26	38	62
**Wines fermented with grape skins**										
GP30	11	89	-	-	-	-	77	23	24	76
GP90	28	72	-	-	-	-	71	29	34	66
**Botrytized wines**										
2P1	21	79	-	-	-	-	70	30	43	57
2P2	14	86	-	-	-	-	70	30	-	-
3P1	27	73	-	-	-	-	69	31	46	54
3P2	24	76	-	-	-	-	66	34	48	52
3P3	15	85	-	-	-	-	71	29	39	61
3P4	22	78	-	-	45	55	61	39	36	64
3P5	14	86	-	-	-	-	77	23	36	64
3P6	18	82	-	-	-	-	75	25	44	56
3P7	70	30	43	57	-	-	-	-	48	52
4P1	35	65	-	-	-	-	-	-	40	60
4P2	24	76	-	-	-	-	-	-	39	61
4P3	20	80	-	-	-	-	61	39	43	57
4P4	32	68	-	-	-	-	-	-	39	61
4P5	20	80	-	-	-	-	68	32	42	58
4P6	36	64	39	61	51	49	-	-	46	54
5P1	38	62	-	-	-	-	-	-	43	57
5P2	24	76	-	-	-	-	-	-	27	73
5P3	26	74	-	-	-	-	-	-	46	54
5P4	21	79	-	-	-	-	65	35	37	63
5P5	20	80	-	-	-	-	58	42	46	54
5P6	25	75	-	-	-	-	64	36	42	58
5P7	22	78	-	-	-	-	70	30	40	60
5P8	43	57	-	-	52	48	-	-	33	67
5P9	42	58	-	-	51	49	-	-	32	68
5P10	41	59	-	-	-	-	74	26	33	67
6P1	29	71	-	-	48	52	71	29	39	61
6P2	25	75	-	-	-	-	-	-	40	60
6P3	54	46	-	-	-	-	-	-	58	42
6P4	15	85	-	-	-	-	-	-	37	63
6P5	23	77	-	-	-	-	75	25	38	62
6P6	27	73	-	-	36	64	71	29	38	62
6P7	30	70	-	-	44	56	80	20	39	61
6P8	81	19	-	-	54	46	58	42	42	58
**Varietal wines**										
F1	77	23	-	-	-	-	73	27	-	-
F2	79	21	-	-	55	45	-	-	42	58
F3	91	9	52	48	58	42	72	28	-	-
M1	-	-	56	44	67	33	-	-	-	-
M2	-	-	48	52	63	37	63	37	-	-
M4	83	17	51	49	59	41	68	32	-	-
M5	73	27	40	60	54	46	-	-	-	-
L1	-	-	38	62	59	41	74	26	-	-
L2	88	12	37	63	-	-	64	36	-	-
L3	50	50	47	53	54	46	-	-	53	47
L4	91	9	41	59	56	44	-	-	-	-
L5	90	10	45	55	56	44	71	29	-	-

2P, 3P, 4P, 5P and 6P are shortcut for “puttony” wines with the corresponding number of tubs was added.

## Abbreviations

GC×GC	comprehensive two-dimensional gas chromatography
RT	retention time
RI	retention index
